# Supplementary material for: Structural basis for recognition of the malaria vaccine candidate Pfs48/45 by a transmission blocking antibody
Source: Nat Commun. 2018 Sep 20;9:3822. doi: 10.1038/s41467-018-06340-9 (PMC6148045; doi:10.1038/s41467-018-06340-9)
Supplement: Supplementary file 1 — Supplementary Information [file 41467_2018_6340_MOESM1_ESM.pdf]

Supplementary Information

Structural basis for recognition of the malaria vaccine candidate Pfs48/45 by a transmission blocking antibody

Lennartz, Brod *et al*

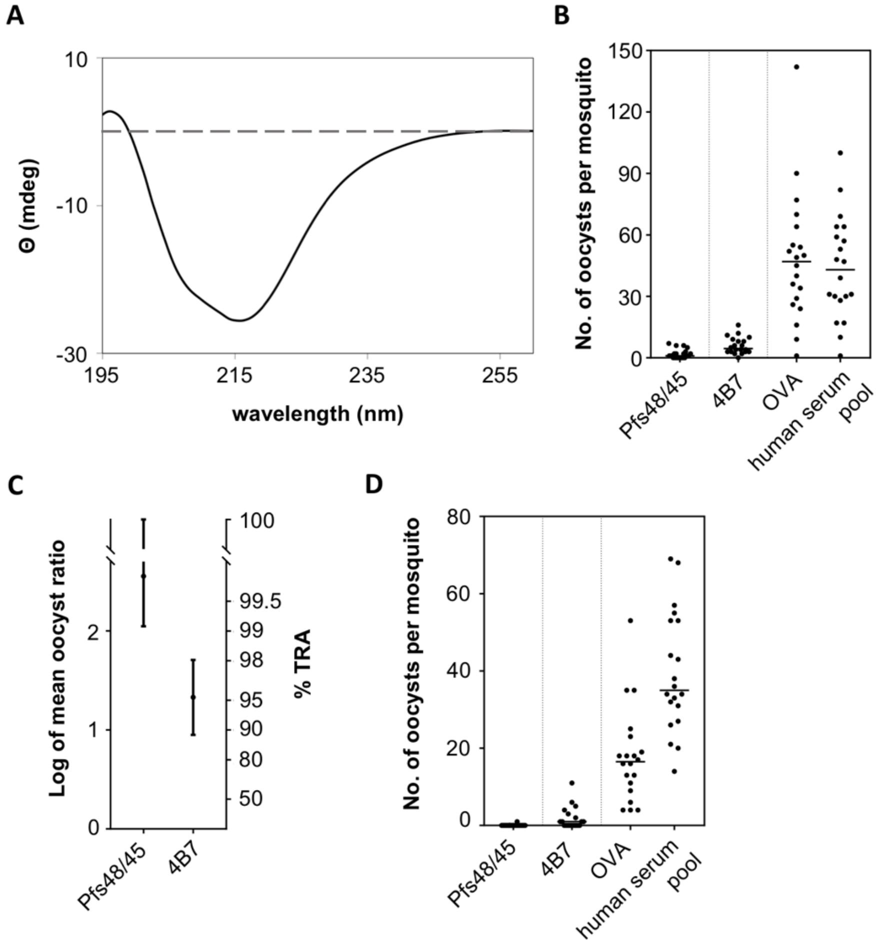

Supplementary Figure 1: SMFA and CD spectroscopy to validate the functionality and fold of full length Pfs48/45

A. Secondary structure analysis of Pfs48/45-FL. CD spectra were recorded at 20°C. Four measurements were averaged and corrected for buffer absorption. **B** Oocyst numbers of SMFA experiments reported in Figure 1. SMFA of purified IgG (750 µg/ml) from mice immunised with Pfs48/45 or OVA were mixed with *P. falciparum* NF54 cultured gametocytes and fed to *A. stephensi* mosquitoes (n = 20 per test group) in SMFA. Midguts were dissected 8 days post-feeding. Data points represent the number of oocysts in individual mosquitoes and the lines show the arithmetic mean. Transmission blocking mAb 4B7 was used as a positive control at a concentration of 94 µg/ml. **C** Results in Figure 1D were confirmed in an independent experiment (n=10). Transmission reducing activity of purified IgG from mice was determined relative to purified IgG from mice immunised with OVA at 750 µg/ml. Transmission blocking mAb 4B7 was used as a positive control at a concentration of 94 µg/ml. Data points show transmission reducing activity (TRA) calculated from the oocyst counts in 20 mosquito midguts. Error bars show 95% CI. SMFA was performed in the absence of human complement. **D** Oocyst numbers of SMFA experiments shown in **C**. Data points represent the number of oocysts in individual mosquitoes (n=20) and the lines show the arithmetic mean.

A

Blocker	Biotinylated mAb															
	Group A								Group B		Group C		no group			
	6A10	1F10	3G3	10D8	2F5	5E4	4C11	6E8	1B12	6E1	3H6	7A7	9D1	10F10	9A6	7A6
6A10	0.40	0.27	0.19	0.25	0.48	0.44	0.16	0.43	2.84	1.90	0.15	0.12	2.43	0.88	0.35	1.56
1F10	0.42	0.17	0.23	0.18	0.51	0.41	0.18	0.48	2.46	2.05	0.13	0.10	0.27	0.82	0.32	1.70
3G3	0.59	0.31	0.29	0.39	0.81	0.65	0.19	0.61	2.05	1.92	0.14	0.09	1.37	0.76	0.40	1.41
10D8	0.60	0.29	0.27	0.11	0.67	0.49	0.23	0.67	1.94	1.81	0.16	0.13	2.16	0.86	0.42	0.43
2F5	0.38	0.23	0.21	0.25	0.51	0.41	0.15	0.41	2.03	1.81	0.15	0.11	1.40	0.76	0.27	1.27
5E4	0.39	0.23	0.19	0.20	0.48	0.38	0.16	0.43	2.49	1.81	0.15	0.13	2.11	0.66	0.35	1.31
4C11	1.01	0.71	0.57	0.80	1.49	1.05	0.32	1.01	2.35	1.90	0.14	0.10	0.82	0.82	0.60	1.42
6E8	0.40	0.24	0.22	0.25	0.44	0.37	0.17	0.47	1.74	1.75	0.15	0.13	1.95	0.75	0.32	1.29
1B12	1.64	2.04	1.73	1.20	2.87	1.64	0.78	1.67	1.31	1.21	0.17	0.13	1.67	0.84	1.11	1.65
6E1	1.41	1.34	1.22	1.15	1.97	1.12	0.68	1.47	1.60	0.14	0.15	0.11	1.31	0.71	1.15	1.54
3H6	1.69	1.38	1.49	1.28	2.73	1.66	0.64	1.53	2.17	2.00	0.09	0.08	1.58	0.98	0.68	1.17
7A7	1.67	1.37	1.43	1.16	2.18	1.45	0.67	1.61	1.87	1.86	0.09	0.08	1.68	0.88	0.63	1.14
9D1	1.86	0.78	1.42	1.73	2.47	1.55	0.64	1.76	1.77	1.91	0.12	0.10	0.18	0.87	0.66	0.90
10F10	1.67	1.80	1.27	1.16	2.07	1.29	0.67	1.65	2.40	1.97	0.17	0.13	2.19	0.36	1.01	1.52
9A6	1.65	1.33	1.23	1.00	2.16	1.40	0.59	1.56	1.91	1.93	0.12	0.10	1.80	1.33	0.31	0.95
7A6	1.65	1.52	1.41	0.61	1.95	1.32	0.65	1.67	1.95	1.85	0.10	0.09	1.44	0.84	0.24	0.32

B

Blocker	Biotinylated mAb	
	32F3	45.1
1B12	2.62	2.59
1F10	2.62	2.67
2F5	2.32	1.99
3G3	2.85	2.28
3H6	2.80	2.33
4C11	2.16	2.01
5E4	2.04	1.97
6A10	2.32	2.06
6E1	2.91	2.93
6E8	2.54	2.69
7A6	2.44	2.22
7A7	2.29	2.18
9A6	2.08	2.01
9D1	2.17	2.06
10D8	1.86	1.86
10F10	1.93	1.75
self	1.18	1.21

C

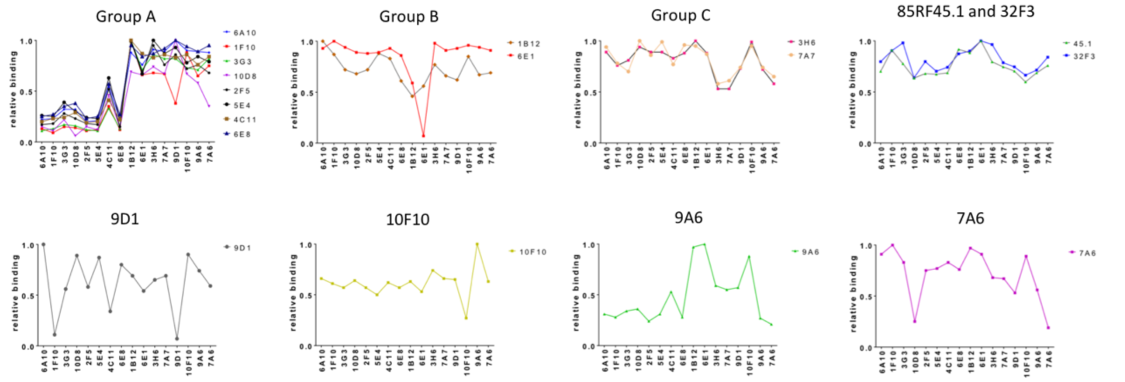

Supplementary Figure 2: Cross-competition ELISA.

A OD_{405} for all combinations of blocking and biotinylated mAbs (average of two technical replicates). **B** OD_{405} of cross competition ELISA of 32F3 and 85RF45.1 against all mAbs in the Pfs48/45 mAb panel (average of two technical replicates). **C** OD_{405} values for all combinations of blocking and biotinylated mAbs were normalised to a value between 1 and 0 to allow plotting on a shared y-axis. Relative binding of each biotinylated mAb calculated this way was plotted against each blocking mAb to visualise competition groups.

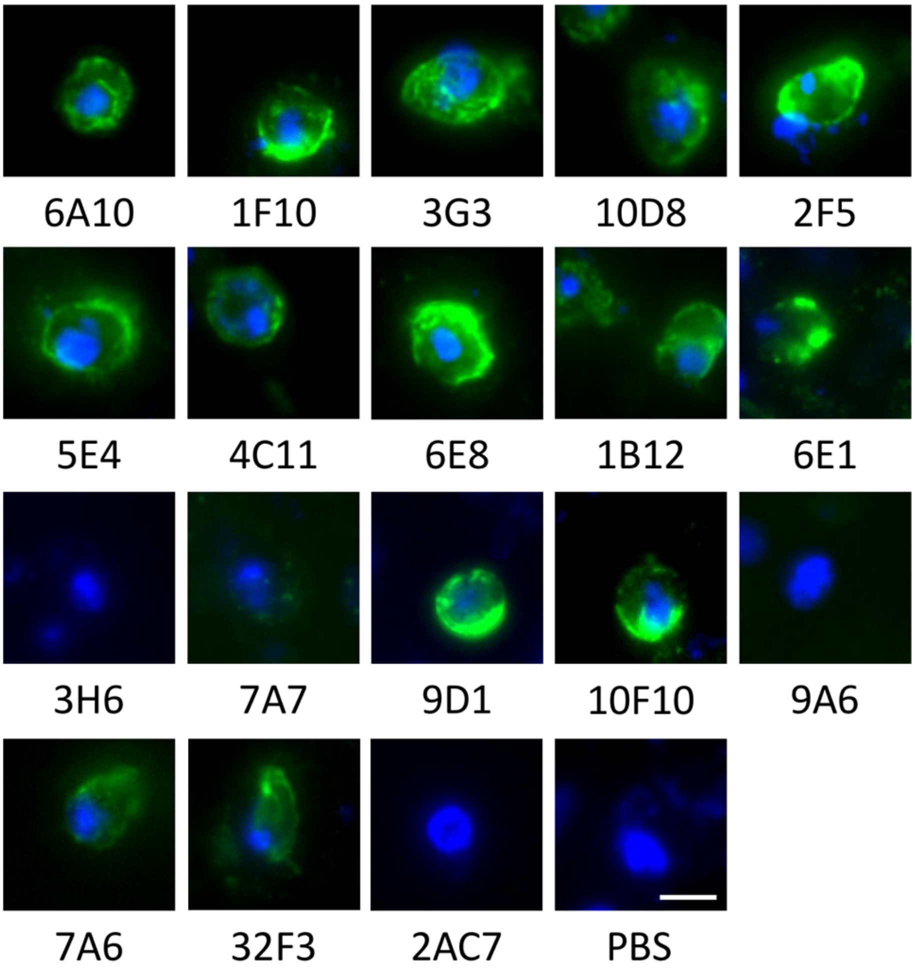

Supplementary Figure 3: Assessing the binding of Pfs48/45 mAbs to gametes

Pfs48/45 mAbs were assessed for their ability to bind to *in vitro* generated *P. falciparum* NF54 gametes. 2AC7 is a negative control that targets PfRH5. A representative image from two technical replicates is shown for each mAb. Images taken at 100x magnification. Scalebar = 2 µm.

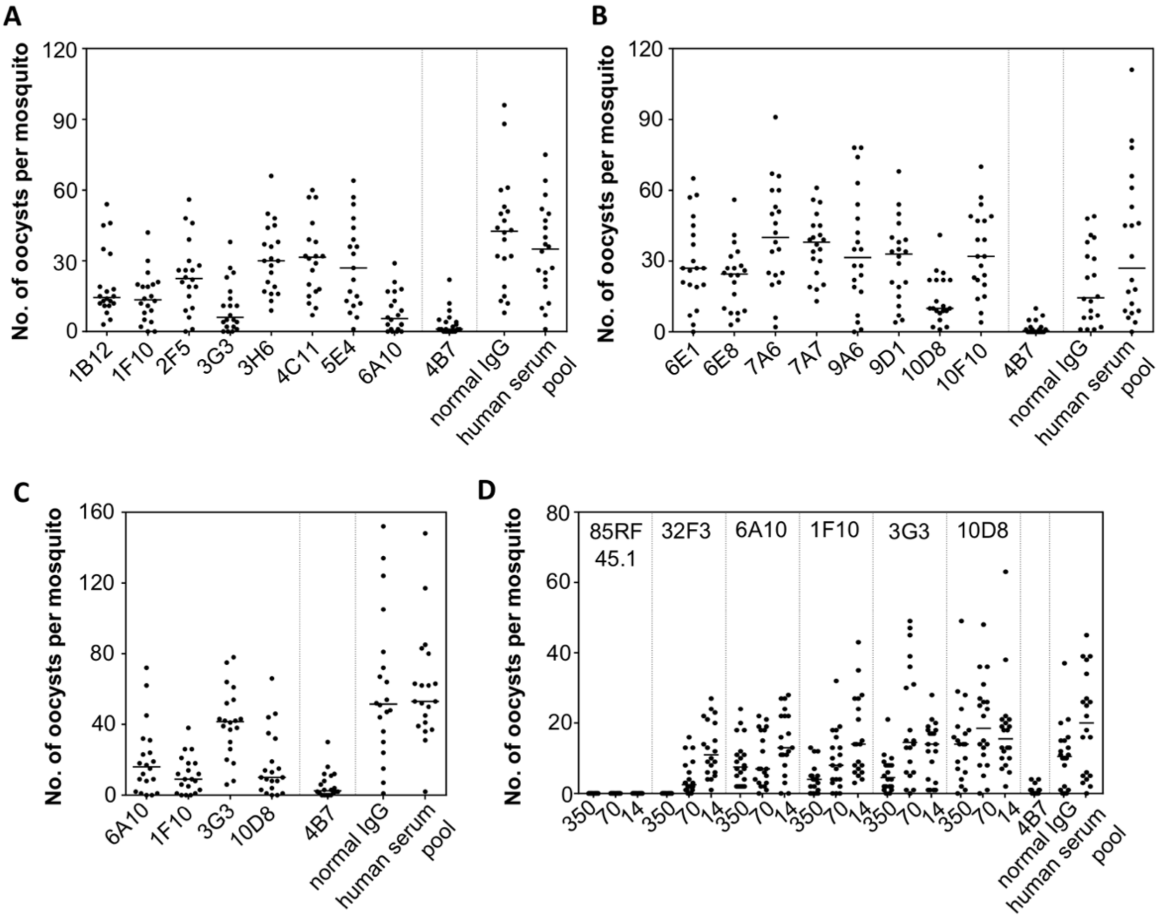

Supplementary Figure 4: Oocyst numbers for SMFA experiments testing newly generated anti Pfs48/45 mAbs and their comparison with previously characterized mAbs.

Monoclonal antibodies were mixed with *P. falciparum* NF54 cultured gametocytes and fed to *A. stephensi* mosquitoes (n = 20 per test group) in SMFA. Midguts were dissected 8 days post-feeding. Data points represent the number of oocysts in individual mosquitoes and the lines show the arithmetic mean. **A and B** SMFA of the anti-Pfs48/45 mAb panel at a concentration of 375 µg/ml. Normal mouse antibody at a concentration of 750 µg/ml was used as a negative control. Transmission blocking mAb 4B7 at a concentration of 94 µg/ml was used as a positive control. **C** Repeat SMFA of the anti-Pfs48/45 mAbs that had shown TRA in the previous SMFA at a concentration of 375 µg/ml. Normal mouse antibody at a concentration of 750 µg/ml was used as a negative control. Transmission blocking mAb 4B7 at a concentration of 94 µg/ml was used as a positive control. **D.** SMFA was performed with the mAbs 85RF45.1, 32F3, 1F10, 3G3, 6A10 and 10D8 at concentrations of 350 µg/ml, 70 µg/ml and 14 µg/ml. Transmission blocking mAb 4B7 was used as a positive control at a concentration of 94 µg/ml. Normal mouse antibody at a concentration of 750 µg/ml was used as a negative control.

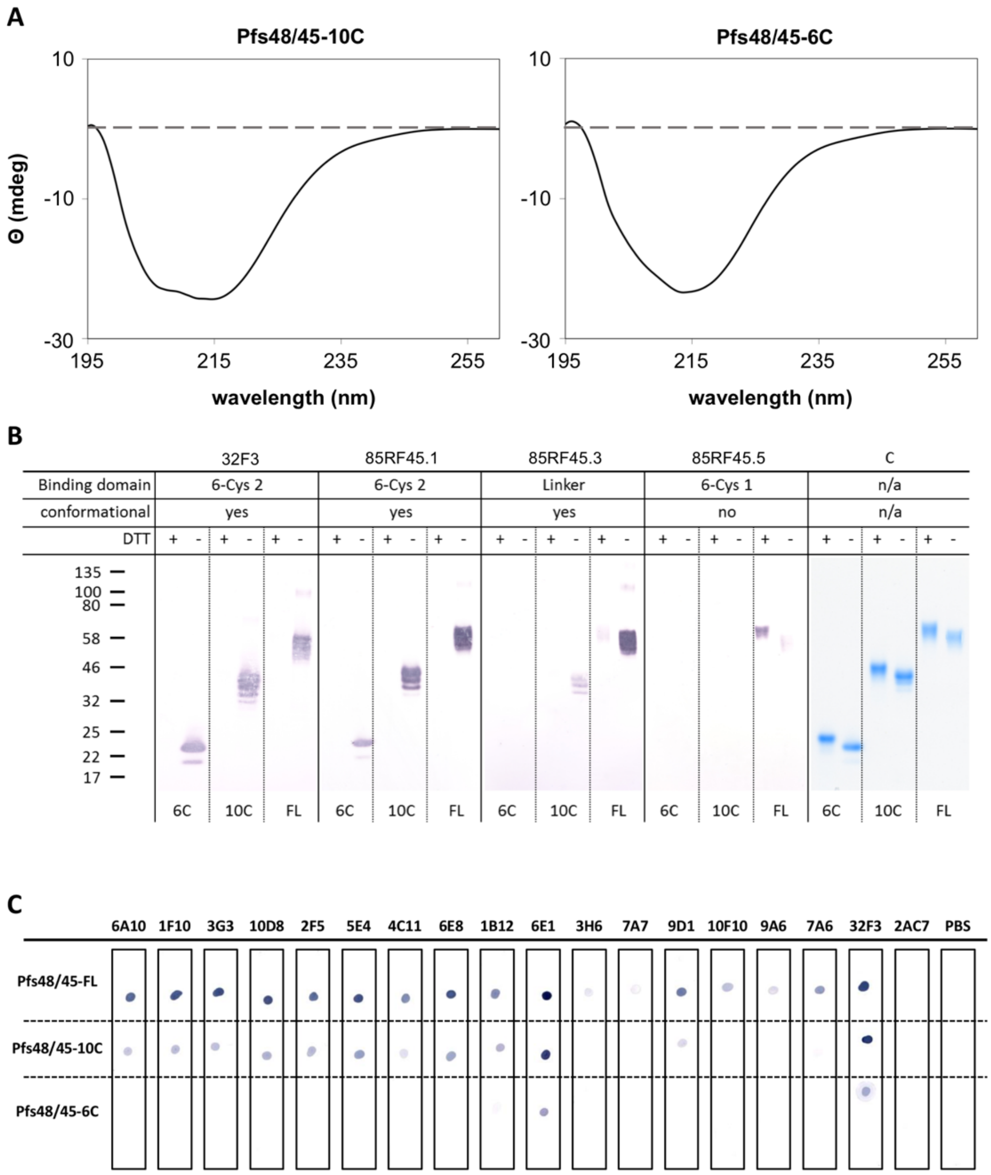

Supplementary Figure 5: CD spectroscopy of Pfs48/45-10C and 6C and their reactivity towards all characterized mAbs

A Secondary structure analysis of Pfs48/45 variants. CD spectra for Pfs48/45-10C and Pfs48/45-6C were recorded at 20°C. Four measurements were averaged and corrected for buffer absorption. **B** Western Blots and Coomassie gel of Pfs48/45-FL, -10C and -6C. Proteins were run on 4-12% Bis-Tris Polyacrylamide gels in the presence and absence of DTT as a reducing agent and then either stained with Coomassie brilliant blue (C) or blotted on nitrocellulose membranes and detected with Pfs48/45 specific monoclonal antibodies (32F3, 85RF45.1, 85RF45.3 and 85RF45.5, at a concentration of 1 µg/ml). **C** Pfs48/45 FL, 10C and 6C were spotted on nitrocellulose membrane strips. Strips were then blocked in 2% BSA in PBS-T and the probed with the panel of anti-Pfs48/45 monoclonal antibodies (at a concentration of 1 µg/ml). 32F3 was used as a positive control. The Rh5 mAb 2AC7 and PBS were used as negative controls.

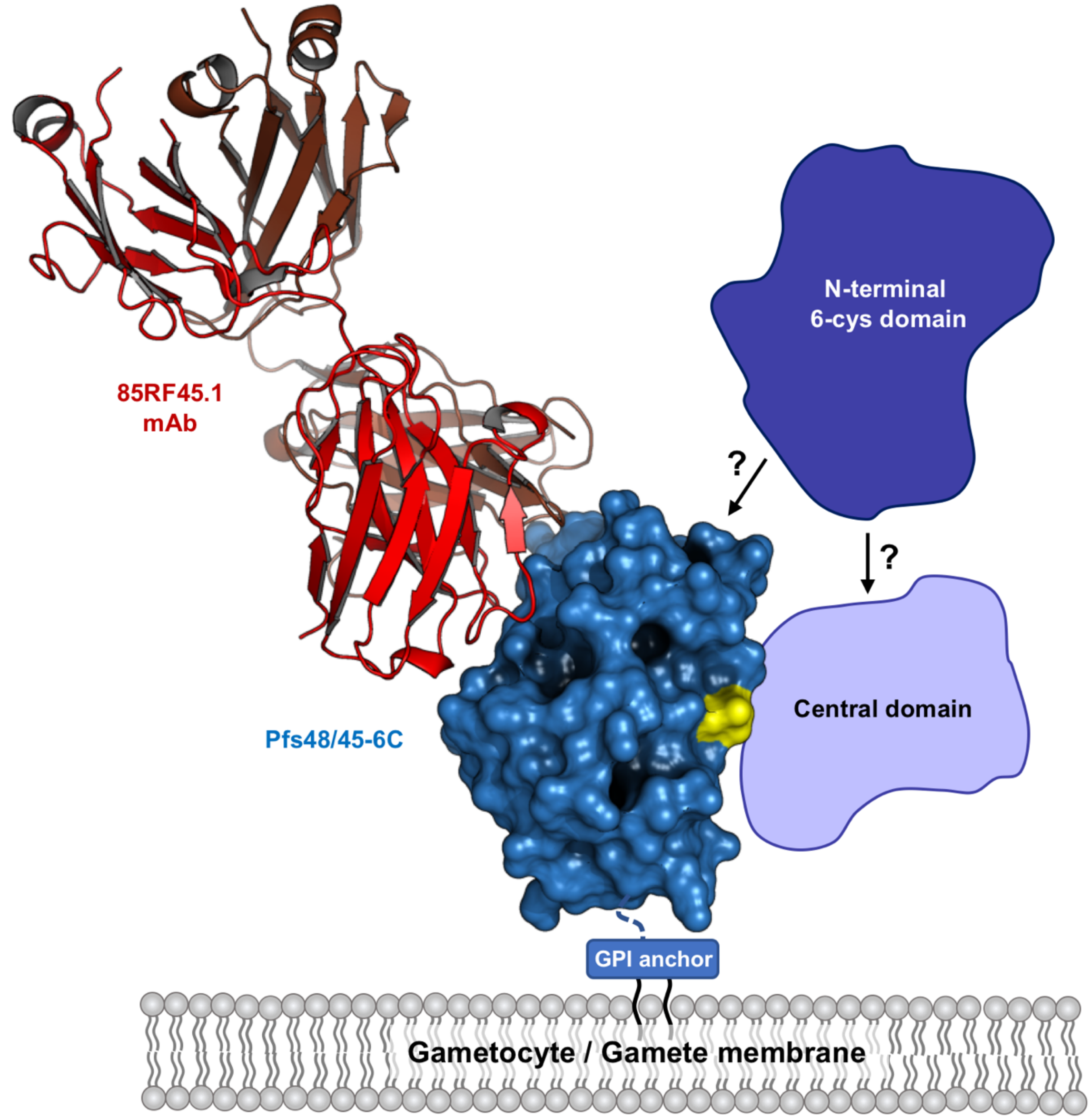

Supplementary Figure 6: Model of how Pfs48/45-6C might be positioned on the gametocyte membrane

The crystal structure of Pfs48/45 is oriented towards the membrane as expected by its C-terminal link to a Glycosylphosphatidylinositol (GPI) anchor (indicated by the dashed line). The locations of the N-terminal and central domains are currently unknown. However, the central domain must emerge from N-terminus (highlighted in yellow) of the C-terminal domain of Pfs48/45 and the N-terminal and central domains must project away from the epitope for 85RF45.1 (red) in full-length Pfs48/45 (see Figures 2 and 3).

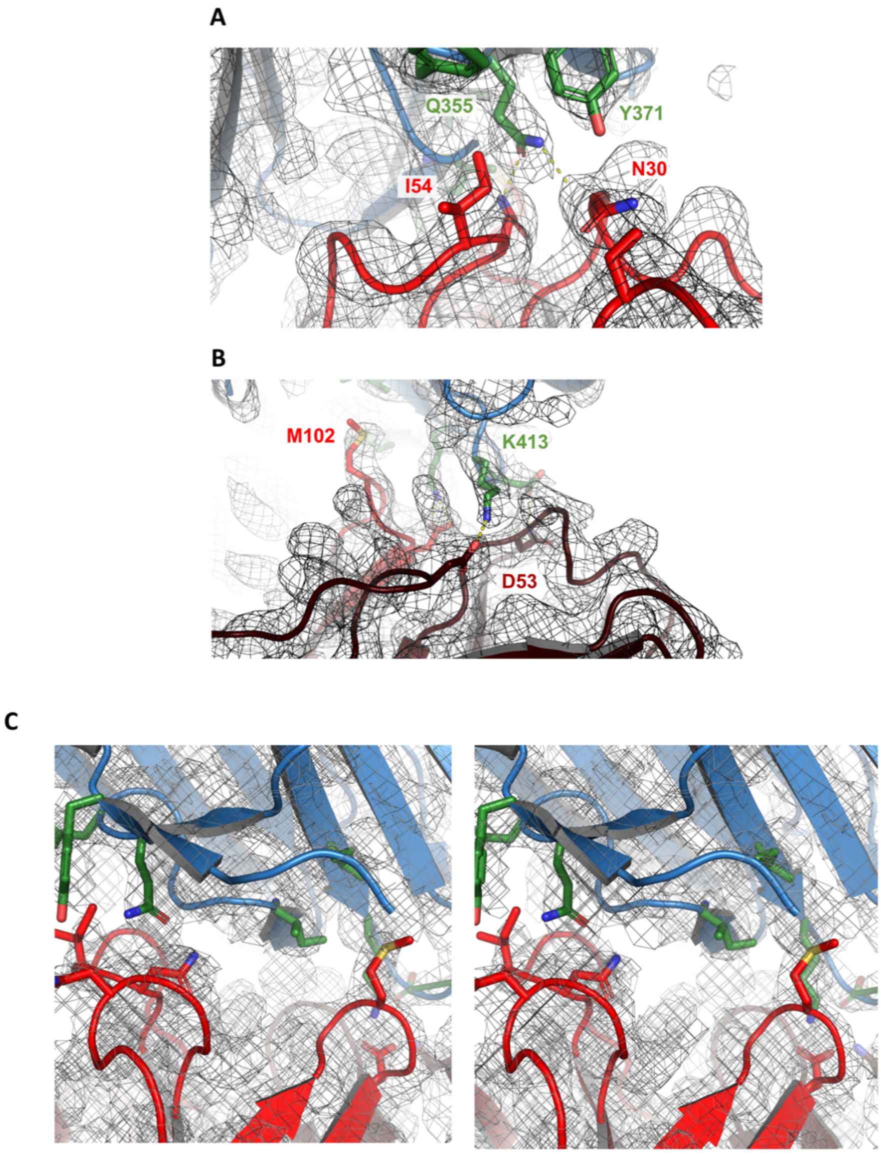

Supplementary Figure 7: Electron density at the Pfs48/45-6C-85RF45.1 Fab interface **A and B** show two different views of the 85RF45.1 Fab – Pfs48/45 Fab interface. **C** shows a stereo view of the electron density at the 85RF45.1 Fab – Pfs48/45 Fab interface. Residues from 85RF45.1 Fab (red/dark red) that interact with Pfs48/45-6C (green) are shown in stick representation and numbered and colored as in Figure 5. The electron density is the refined 2Fo-Fc electron density shown at 1σ. Hydrogen bonds are indicated by dashed lines

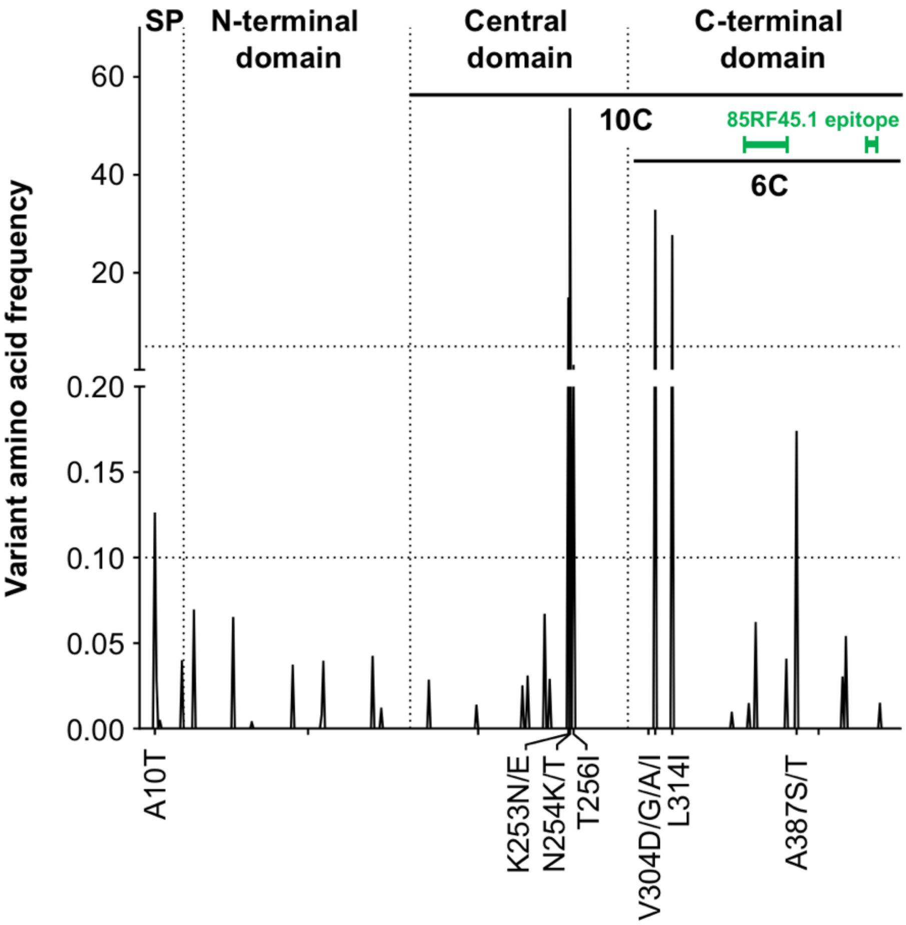

Supplementary Figure 8: Amino acids polymorphisms among Pfs48/45 sequences in the Pf3k database.

Variant amino acid frequency was calculated as the alternative allele depth divided by the total allele depth for all single-nucleotide polymorphisms (SNPs) coding an amino acid change within Pfs48/45 in Pf3k release 3 (>2,400 sequences). Where multiple SNPs caused a change in the same amino acid, their frequencies were added together. Variant amino acid frequency was then plotted against the amino acid position within Pfs48/45 and amino acid changes with a frequency of above 0.1% were annotated. Green bars indicate the location of the 85RF45.1 epitope.

***Supplementary Table* 1. List of contacts between 85RF45.1 Fab and Pfs48/45-6C**

Colors correspond to the 85RF45.1 heavy and light chain as coloured in Figures 4 and 5 of the main text

**85RF45.1 Fab**		**Pfs48/45-6C**		
**Residue**	**Group**	**Residue**	**Group**	**Type of Interaction**
**Asn30**	Sidechain NH_2	Tyr371	Sidechain OH	Hydrogen bond
**Asn53**	Sidechain NH_2	Gln355	Sidechain O	Hydrogen bond
**Ile54**	Sidechain	Tyr371	Sidechain	Hydrophobic
**Ile54**	Sidechain	Phe354	Sidechain	Hydrophobic
**Met102**	Sidechain	Ile349	Sidechain	Hydrophobic
**Met102**	Sidechain	Ile411	Sidechain	Hydrophobic
**Met102**	Sidechain	Lys413	Sidechain	Hydrophobic
**Asp104**	Sidechain	Lys413	Sidechain NH_3	Hydrogen bond
**Asn32**	Sidechain NH_2	Asp415	Sidechain O	Hydrogen bond
**Asp52**	Sidechain O	Lys416	Sidechain NH_3	Hydrogen bond
